# Perspectives of Adolescents and Young Adults With Inflammatory Bowel Disease on a Biopsychosocial Transition Intervention: Qualitative Interview Study

**DOI:** 10.2196/64618

**Published:** 2025-04-02

**Authors:** Brooke Allemang, Ashleigh Miatello, Mira Browne, Melanie Barwick, Pranshu Maini, Joshua Eszczuk, Chetan Pandit, Tandeep Sadhra, Laura Forhan, Natasha Bollegala, Nancy Fu, Kate Lee, Emily Dekker, Irina Nistor, Sara Ahola Kohut, Laurie Keefer, Anne Marie Griffiths, Thomas D Walters, Samantha Micsinszki, David R Mack, Sally Lawrence, Karen I Kroeker, Jacqueline de Guzman, Aalia Tausif, Claudia Tersigni, Samantha J Anthony, Eric I Benchimol

**Affiliations:** 1 Child Health Evaluative Sciences, SickKids Research Institute, The Hospital for Sick Children, Toronto, ON, Canada; 2 Faculty of Health Sciences, McMaster University, Hamilton, ON, Canada; 3 Department of Psychiatry, University of Toronto, Toronto, ON, Canada; 4 Dalla Lana School of Public Health, University of Toronto, Toronto, ON, Canada; 5 Faculty of Kinesiology, University of Alberta, Edmonton, AB, Canada; 6 ETH Zurich, Zurich, Switzerland; 7 Faculty of Health, York University, Toronto, ON, Canada; 8 Department of Molecular and Cellular Biology, University of Guelph, Guelph, ON, Canada; 9 Division of Gastroenterology, Women’s College Hospital, Toronto, ON, Canada; 10 Department of Medicine, University of Toronto, Toronto, ON, Canada; 11 Division of Gastroenterology, Department of Medicine, University of British Columbia, Vancouver, BC, Canada; 12 Crohn’s and Colitis Canada, Toronto, ON, Canada; 13 SickKids Inflammatory Bowel Disease Centre, Division of Gastroenterology, Hepatology and Nutrition, The Hospital for Sick Children, 555 University Avenue, Toronto, ON, M5G 1X8, Canada, 1 416-813-1500; 14 Division of Gastroenterology, Icahn School of Medicine at Mount Sinai, New York, NY, United States; 15 Department of Paediatrics and Institute of Health Policy, Management and Evaluation, University of Toronto, 555 University Avenue, Toronto, ON, Canada; 16 CanChild Centre for Childhood Disability Research and the School of Rehabilitation Science, McMaster University, Hamilton, ON, Canada; 17 CHEO Inflammatory Bowel Disease Centre, Division of Gastroenterology, Hepatology and Nutrition, Children's Hospital of Eastern Ontario, Ottawa, ON, Canada; 18 Department of Pediatrics, University of Ottawa, Ottawa, ON, Canada; 19 Division of Gastroenterology, Hepatology and Nutrition, BC Children’s Hospital, Vancouver, BC, Canada; 20 Department of Pediatrics, University of British Columbia, Vancouver, BC, Canada; 21 Division of Gastroenterology, Department of Medicine, University of Alberta, Edmonton, AB, Canada; 22 Factor-Inwentash Faculty of Social Work, University of Toronto, Toronto, ON, Canada; 23 ICES, Toronto, ON, Canada

**Keywords:** gastroenterology, inflammatory bowel disease, biopsychosocial, patient-oriented research, transition to adult care, qualitative methods, young adults, qualitative, adolescents, patient perspectives, Crohn's disease

## Abstract

**Background:**

The transition from pediatric to adult health care marks a complex and pivotal process for adolescents and young adults with inflammatory bowel disease (IBD). This group requires support regarding disease self-management, skill development, and system navigation in preparation for transition. Evidence-based interventions are needed to promote optimal health and psychosocial outcomes for adolescents and young adults with IBD during this period.

**Objective:**

A qualitative study embedded within a randomized controlled trial was conducted to evaluate the perceived impact of a biopsychosocial transition intervention on the transition experiences of adolescents and young adults, their views on the intervention, and recommendations for future care.

**Methods:**

This patient-oriented research study used a qualitative descriptive design. Virtual semistructured interviews were held with 21 adolescents and young adults with IBD (16‐18 y) enrolled in the randomized controlled trial (intervention arm n=11 and control arm n=10). Interviews were audio-recorded, transcribed, and analyzed using an inductive approach to reflexive thematic analysis. Five members of a Youth Advisory Panel with lived experience of IBD collaborated throughout data analysis, interpretation, and the presentation of findings.

**Results:**

We constructed three themes through our analysis: (1) making meaning of transitions in care; (2) perceptions and impact of the biopsychosocial transition intervention; and (3) considerations for future transition care, including the importance of individualized support.

**Conclusions:**

Our findings illustrate the importance of relationships and the impact of a biopsychosocial intervention on adolescents’ and young adults’ confidence, knowledge, and self-management skills during transition. The results, which indicate the criticality of tailoring transition supports according to adolescents’ and young adults’ preferences and characteristics, will be used to refine the biopsychosocial intervention before it can be scaled and spread.

## Introduction

The transition from the pediatric to the adult health care system can be a complex period for adolescents and young adults with inflammatory bowel disease (IBD) and their families [1]. In adult care, adolescents and young adults are expected to self-advocate, communicate with providers, understand their health history, and manage their health with greater independence [2,3]. However, service disruptions at the pediatric-adult juncture, shifts in parental involvement, a lack of readiness for transition, and differing treatment philosophies and models of care can complicate the transition process [1,4]. While the transition from pediatric to adult care for adolescents and young adults with IBD has been well studied and identified as a priority area for policy and program development [5-7], evidence-based transition interventions that account for the priorities of adolescents and young adults are needed.

Adolescents and young adults with IBD face a series of challenges around the transition from pediatric to adult care [1]. Psychosocial stressors are prominent at this stage of life, including the emergence or worsening of mental health conditions, changing roles within the family, and co-occurring transitions in the areas of employment, education, and living [8]. Termination of longstanding relationships with pediatric providers and expectations for autonomous management of one’s health condition following a transfer out of pediatric care can exacerbate the challenges associated with this period [1,9]. Additionally, this group is susceptible to poor health outcomes and high health care costs should the transition from pediatric to adult care be disjointed [10-12]. Therefore, a purposeful, coordinated, and planned transition from the pediatric to the adult health care system is required to ensure adolescents and young adults with IBD can achieve the best possible health and psychosocial outcomes during a complex developmental period [13].

Transition interventions for adolescents and young adults with IBD to date have typically included joint pediatric-adult clinic visits, face-to-face education about disease processes and self-management, and meetings with allied health professionals focused on self-efficacy and goal setting [14]. However, transition intervention components are highly variable and limited research has focused on adolescents’ and young adults’ perspectives of the most valuable and well-received aspects of such interventions [14]. Further, there is no level-one evidence (eg, randomized controlled trials [RCTs]) for any intervention in transition in IBD [15]. To improve the standards for health care delivery and transition care across Canada for adolescents and young adults with IBD, a biopsychosocial transition intervention was developed and is currently being evaluated in a type 1 hybrid effectiveness-implementation trial (ClinicalTrials.gov NCT05221281) [16]. While various functional and implementation outcomes are being assessed within the RCT using standardized measures, qualitative research can elucidate adolescents’ and young adults’ needs and experiences of the intervention to support the translation of findings into practice [17]. Thus, the objectives of this study were to explore adolescents’ and young adults’ transition experiences, perceptions of the transition intervention, and recommendations for future transition care to inform the refinement of the intervention.

## Methods

### Study Design and Population

**Figure 1.**
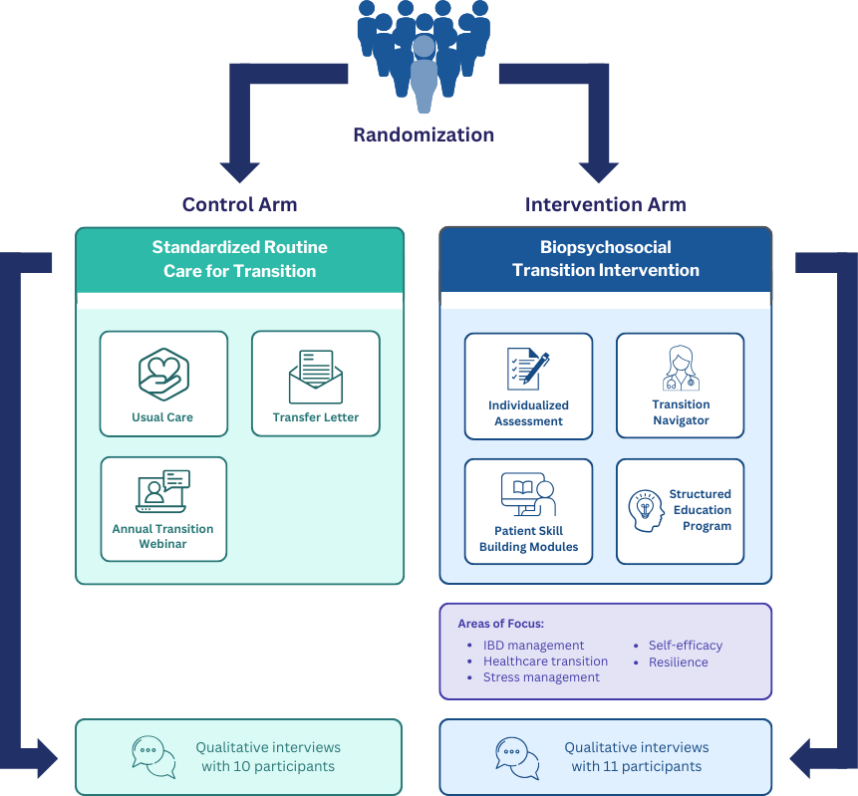
Overview of RCT design. IBD: inflammatory bowel disease; RCT: randomized controlled trial.

This patient-oriented research study [18] adopted a qualitative descriptive design [19] to understand the needs and experiences of adolescents and young adults with IBD during the transition while concurrently assessing the perceived impact and acceptability of the biopsychosocial transition intervention. It was embedded within an ongoing multicenter RCT evaluating the effectiveness of the transition intervention among adolescents and young adults with IBD [16]. A qualitative descriptive design was used to explore the perspectives of adolescents and young adults in addition to the outcomes being assessed in the RCT [16,19]. The intervention consisted of (1) an individualized assessment, (2) a transition navigator, (3) patient skill-building delivered via online modules, and (4) a structured education program (see Figure 1 for an overview of the RCT design) [16]. Half of the RCT participants were randomized to the biopsychosocial intervention, and half received a standardized version of routine care for transition [16]. Adolescents and young adults in the intervention arm received support from a transition navigator. They had access to online skill-building activities and an online education program with topics focused on IBD management, resilience, health care transition, self-efficacy, and stress management for the duration of the study [16]. Though the RCT has 3 study sites at Canadian tertiary care centers, this study explored the perspectives of adolescents and young adults at the study site, which has the largest cohort of participants and has participants who had access to the intervention for the longest duration.

In embracing a patient-oriented research approach [18], the study investigators collaborated with a project-specific Youth Advisory Panel (YAP) consisting of 5 patient partners with diverse backgrounds, expertise, and lived experience with IBD. YAP members were recruited from an existing national IBD advisory council. The national IBD advisory council was involved in a previous qualitative study conducted by our team [20]. Subsequently, select advisory panel members expressed the desire to become involved in this study to further explore and contribute to the qualitative findings. The YAP consisted of 3 male and 2 female members aged 18‐30 years from across Canada, thereby increasing the study’s transferability with enhanced diversity of input and representation of health care experiences. The YAP members actively contributed to data analysis, interpretation, presentation of results, and preparing this article, helping align research findings with patient-identified priorities [18]. This collaborative partnership and bidirectional flow of knowledge helped cultivate a sense of ownership in members while capturing nuanced insights often overlooked in traditional research paradigms. YAP members were supported and mentored by 2 study team members (BA and AM) throughout the process. Study investigators and YAP members frequently corresponded via email. They held virtual weekly or biweekly progress meetings over 3 months (March-May 2024) with flexible agendas to promote clarity of purpose, efficient use of time, and active participation during meetings.

### Eligibility and Sampling

Study procedures are available in a prior publication [20]. Briefly, eligible qualitative participants were the following: (1) aged 16‐20 years, (2) English-speaking, (3) diagnosed with IBD, (4) enrolled in the RCT at the SickKids study site for at least six months, and (5) capable of providing informed consent. Purposive sampling [21] was used to select a clinically and demographically diverse sample of adolescents and young adults in the intervention and control arms, with varying levels of engagement with the intervention. We discontinued recruitment once we gleaned a breadth and depth of relevant information from participants to answer our study questions [22].

### Data Collection

A female qualitative researcher with a social work background conducted individual interviews with adolescents and young adults using an institutional account of Microsoft Teams (Microsoft Corp). A semistructured interview guide, developed with YAP members and an interdisciplinary team of IBD clinicians and researchers, was used. Interview questions focused on adolescents’ and young adults’ experiences preparing for or undergoing health care transitions, perceptions of the biopsychosocial intervention, and recommendations for future transition care in IBD. Consent was obtained, and interviews were digitally recorded, transcribed, verified, and anonymized before analysis. Reflective memos were used during data analysis to highlight striking patterns and impressions of the interview data [23]. Participants received a US $20 electronic gift card and 1 volunteer hour for completing an interview.

### Data Analysis

An inductive approach to reflexive thematic analysis [23,24] was used to analyze the data. Interview transcripts were reviewed by 2 coders (BA and M Browne) who reflected on their positionality, assumptions, and social locations in reference to the data. The coders then engaged in line-by-line coding inductively, designating codes to portions of interview text to categorize shared ideas [23,24]. They met consistently during the coding process to consider their perceptions of the data, important codes, and emerging patterns and to begin collectively making sense of the data [23]. Codes were grouped into themes to capture the meaning and key concepts within the data, and relationships between codes and themes were examined using mind maps. YAP members independently reviewed the list of codes and preliminary mind maps. YAP members shared their opinions on organizing concepts, developing themes and figures, and determining which ideas were most important to convey synchronously at online team meetings using the Microsoft Teams platform and asynchronously using collaborative documents. The preliminary themes, codeveloped with the YAP, were presented to the larger team for input, refined, and final themes were named and consolidated through discussion. Analysis was conducted using NVivo (version 14; Lumivero).

### Ethical Considerations

Institutional approval for this study was obtained from The Hospital for Sick Children (SickKids) Research Ethics Board (REB #1000078476). Informed consent was obtained for each participant in the RCT and prior to enrollment in this substudy.

## Results

### Overview

A total of 21 adolescents and young adults were interviewed between May and September 2023 (intervention arm=11 and control arm=10). Participants were aged between 16 and 18 years, primarily female, and at different stages of health care transition. The demographic and clinical characteristics of the participants are presented in Table 1. Participant identifiers beginning with “C” denote control arm participants, and those beginning with “I” indicate intervention arm participants in the results. We constructed three themes through our analysis: (1) making meaning of transitions in care; (2) perceptions and impact of the biopsychosocial transition intervention; and (3) considerations for future transition care, including the importance of tailored supports.

**Table 1.** Participant characteristics (N=21).

				Values, n (%)
Demographic characteristics				
	Gender			
		Female		13 (61.9)
		Male		7 (33.3)
		Nonbinary		1 (4.8)
	Age (years)			
		16		2 (9.5)
		17		15 (71.4)
		18		4 (19.1)
	Ethnicity			
		Black		4 (19)
		South Asian		6 (28.6)
		White		9 (42.9)
		Other or multiracial		2 (9.5)
	Immigration status (participant)			
		Born in Canada		19 (90.5)
		Immigrated to Canada		2 (9.5)
	Immigration status (parents)			
		Born in Canada		7 (33.3)
		Immigrated to Canada		14 (66.7)
	Sexual orientation^a^			
		Bisexual		2 (9.5)
		Gay or lesbian		2 (9.5)
		Heterosexual or straight		15 (71.5)
		Other		2 (9.5)
	Household income of family of origin (US $)			
		0‐49,999		1 (4.8)
		50,000‐99,999		1 (4.8)
		100,000‐149,999		3 (14.3)
		150,000‐199,000		3 (14.3)
		200,000+		3 (14.3)
		I do not know		10 (47.5)
	Study arm			
		Control arm		10 (47.6)
		Intervention arm		11 (52.4)
	Highest level of parental education			
		High school		1 (4.8)
		Some postsecondary		1 (4.8)
		Graduated postsecondary		19 (90.4)
	Vocational status^b^			
		Employed (full or part-time)		6 (28.6)
		High school student		18 (85.7)
		Postsecondary student		1 (4.8)
		Not currently in school or employed		0 (0)
Clinical characteristics				
	Diagnosis type			
		Crohn disease		15 (71.4)
		Inflammatory bowel disease type		
			IBDU^c^	2 (9.5)
			UC^d^	4 (19.1)
	Age at diagnosis (years)			
		≤5		2 (9.5)
		6‐12		5 (23.8)
		13‐17.9		14 (66.7)
	Family history of IBD^e^			
		Yes		7 (33.3)
		No		14 (66.7)
	Transferred to adult gastroenterologist at the time of interview?			
		Yes		4 (19.1)
		No		17 (80.9)

a Response categories based on participants’ language.

b Multiple response options were possible.

c IBDU: inflammatory bowel disease type unclassified.

d UC: ulcerative colitis.

e IBD: inflammatory bowel disease.

### Theme #1: Making Meaning of Transitions in Care

#### Overview

Adolescents and young adults expressed a range of reflections on the meaning of the transition from pediatric to adult health care. Their conceptualizations of this transition ranged from “switching doctors” to more existential thoughts about the health care transition, marking the loss of childhood. The meanings ascribed to health care transitions appeared to influence adolescents’ and young adults’ feelings about entering adult care and their overall readiness to engage in self-care tasks with greater autonomy.

On one end of the spectrum, adolescents and young adults described the transition to adult care as “an inevitability and a necessity” [C-036] once they were aged 18 years. Participants with this view seemed to understand transition as moving from one specialist to the next, with expectations that care would be delivered similarly. Those who conceptualized this change as primarily related to switching providers without ascribing a deeper philosophical meaning to transition tended to express minimal concerns or worries about entering the adult system. Many of these participants had well-managed IBD or family members (eg, parents or siblings) with IBD who were already receiving treatment in the adult system. As described by 1 participant, “For me, it’s not a big deal, you’re just switching doctors. And it’ll just be the same thing over there anyway, so I’m not really stressed out about it.” [C-001]. Importantly, however, those with this mindset had not yet transferred or been exposed to the adult care environment.

Others reflected that the transition to adult health care aligned with the launch from adolescence to young adulthood. Many adolescents and young adults viewed the health care transition as a signal that they were now “grownups” [I-039] who would be required to assume new roles and responsibilities within and outside their IBD management. Some adolescents and young adults felt the transition allowed them to reflect on their growth and development. There was an overarching sense of hope for the future, while, at times, this was coupled with feelings of sadness about the loss of relationships with familiar pediatric providers: “I mean, it’s a sign of maturity and that I’m growing. It’s kind of sad in that way, but you know, it’s a part of life.” [C-031]. Most participants accepted the transition and described it as a period in which they would take greater initiative, advocate for themselves, learn more about their health history, and plan their appointments. One participant shared:

I look at [transition] as me having to know a lot about myself, especially where I am with my health. At the end of the day, my mother has to stop bringing me to the appointments, I can't bring her all the time, right? Because it’s that independent thing that comes in. So I think it’s just a learning experience about myself, learning about where I am with my health, knowing everything that I need to know for myself so I don't have to depend on anybody else to know it for me.

[I-021]

Most participants felt motivated, empowered to take ownership of their care, and even excited to enter a new environment, mainly due to the reputations of the adult IBD clinics or specialists and their confidence in their ability to self-advocate. However, a few adolescents and young adults expressed some hesitance about moving into adult care due to the perceived expectations of adult providers: “While new things are exciting, they’re also unknown and unknown things are scary. [My adult team] will have expectations for grownups, but I don’t really feel like a grownup” [I-039]. The following subtheme delves into the role of relationships between adolescents and young adults and their support networks.

#### Relationality and Transitions to Adult Care

Trusting relationships with health care providers, family members, and peers emerged as transition facilitators for adolescents and young adults. When participants had strong and supportive relationships with their pediatric health care providers, they expressed confidence in their adult IBD providers’ ability to meet their needs: “I have lots of trust in my pediatric doctor, so if he recommended me to this one then this one must be pretty great.” [C-014]. Adolescents and young adults outlined their hopes for developing trust with their new providers in adult care and shared examples of factors promoting engagement with clinicians. These included providers offering clear and detailed responses to adolescents’ and young adults’ questions, adopting a nonjudgmental stance, being transparent about treatment plans, offering adolescents and young adults choices, listening intently to their concerns, and asking for adolescents’ and young adults’ input on health-related decisions.

Adolescents and young adults receiving the biopsychosocial intervention described their relationship with the transition navigator as being one of the most beneficial aspects of this study. Many felt the navigator played a key role in preparing them for the transition by serving as a point of contact for any questions or concerns and “checking in” with adolescents and young adults, particularly between their final pediatric and first adult appointments. Adolescents and young adults described the transition navigator as demonstrating genuine care for their well-being, offering support with the transition to postsecondary education, encouraging the development of self-management skills, and providing anticipatory guidance about what to expect in adult care. “[My transition navigator] made that effort to make me feel like she’s not just a transition navigator, she’s a person that actually cares about me.” [I-015]. The quality of their relationship with the transition navigator also prompted some adolescents and young adults to open up about their emotions and mental health. In one case, this led to a participant receiving a diagnosis for longstanding anxiety symptoms and subsequently accessing mental health supports:

Before I got diagnosed with generalized anxiety, I talked to my transition navigator about how I was feeling and how I didn't really know how to cope. She helped me make the decision to go see a doctor about it to see if [I] could get prescribed medication or get some sort of diagnosis. Even though I was kind of on edge about going to a doctor for something that sounded so silly in my brain, she helped me calm myself about it.

[I-016]

Lastly, adolescents’ and young adults’ relationships with family members and peers facilitated the acquisition of self-management skills and enhanced confidence in preparation for transition. They described the pivotal roles their parents played in helping them cope with IBD and how they are taking more responsibility for specific tasks over time:

My parents have always been supportive and on top of it, maybe even a little more than I have. Because at first, I was in denial, I was like, “no, my IBD’s not that bad”. But they were keeping me on top of my medication and stuff, especially when I was younger, my mom helped me a lot with that. I've become a little more independent now and I'm able to take medication on my own.

[I-020]

Many outlined the value of having parents, siblings, and extended family members offering emotional support (eg, listening, reassuring, or encouraging), instrumental support (eg, teaching how to refill prescriptions), and advice as they approach the transition to adult care. Parents often served as mentors to adolescents and young adults, coaching them to assume greater responsibility for their care and IBD management incrementally. Most participants were aware their roles would change in adult care and were working with parents to practice skills and negotiate what these new roles would be like posttransfer.

The transition experience appeared to be constructed in relation to others. Trusting relationships with health care providers, family members, and peers were found to promote confidence and readiness to engage in the tasks required to manage IBD care during transition. Adolescents’ and young adults’ ability to communicate their needs openly to others (and feel validated by them) was a priority for participants, and supportive relationships helped facilitate this.

### Theme #2: “It Gets Me Ready to Start Doing Things on My Own”: Perceptions and Impact of the Biopsychosocial Transition Intervention

#### Overview

**Figure 2.**
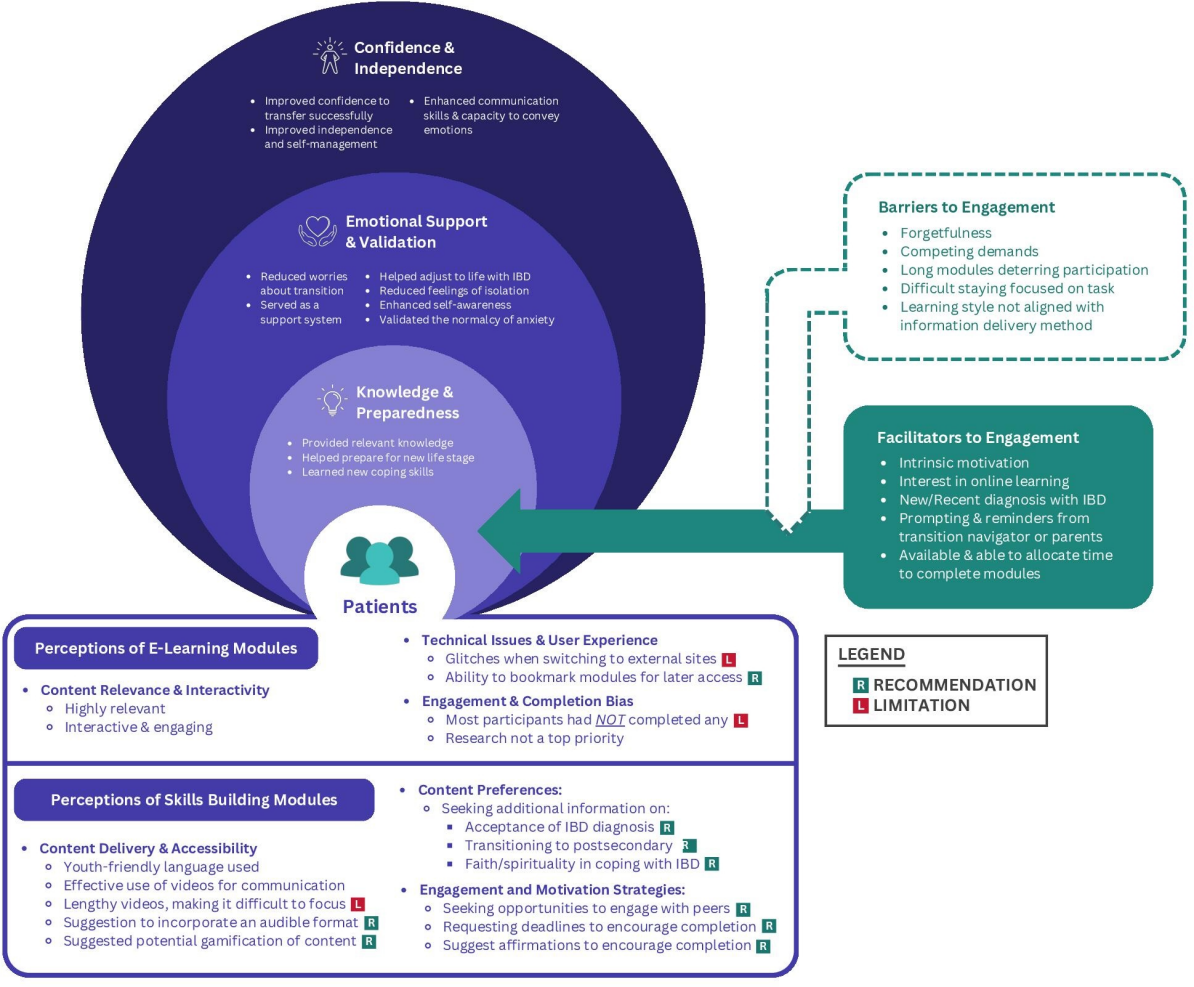
Impact and perceptions of the biopsychosocial transition intervention. IBD: inflammatory bowel disease.

A combination of adolescents’ and young adults’ communication preferences, personality characteristics (eg, curiosity, motivation, or social), learning styles, and sources of support influenced their uptake of and perspectives on the intervention. Participants’ perceptions of the intervention’s impact fell into three categories: (1) knowledge and preparedness, (2) emotional support and validation, and (3) confidence and independence. Additionally, barriers and facilitators to engagement with the intervention were described by adolescents and young adults (Figure 2).

#### Knowledge and Preparedness

Adolescents’ and young adults’ experiences of the intervention indicated that acquiring knowledge helped foster a sense of agency and supported their transition readiness. Core components of the transition intervention helped adolescents and young adults envision their transition journeys while arming them with the competencies required to succeed in adult care and life. Participants receiving the intervention expressed positive sentiments about their experiences in the trial, noting that it catalyzed personal growth and empowerment. Shifting from a mindset of passive acceptance to one of proactive engagement in navigating the complexities of transition, many participants, with curious mindsets, described applying newly acquired skills and knowledge in their daily lives to improve aspects of pain management, communication, and self-regulation:

The transition from pediatric care to adult healthcare is one that’s not easy, [but] there are things that you can learn in order to be more successful during transition with respect to being an adult. I think it’s really just using your skills, engaging in things like mindfulness, and building your confidence so that you can communicate with your healthcare team as best as you can.

[I-019]

A few participants even considered the newfound sense of agency to extend beyond health care transitions to encompass broader life transitions, such as education, career choices, and relationships:

Before I started this, I barely even knew what transition was! It was very foreign to me and it was almost scary because I didn't know what to expect. Especially in addition to going from high school to university, there was just a lot of stuff going on in relation to transition. So having these modules, having this information is something that has definitely helped me in that sense.

[I-007]

#### Emotional Support and Validation

**Figure 3.**
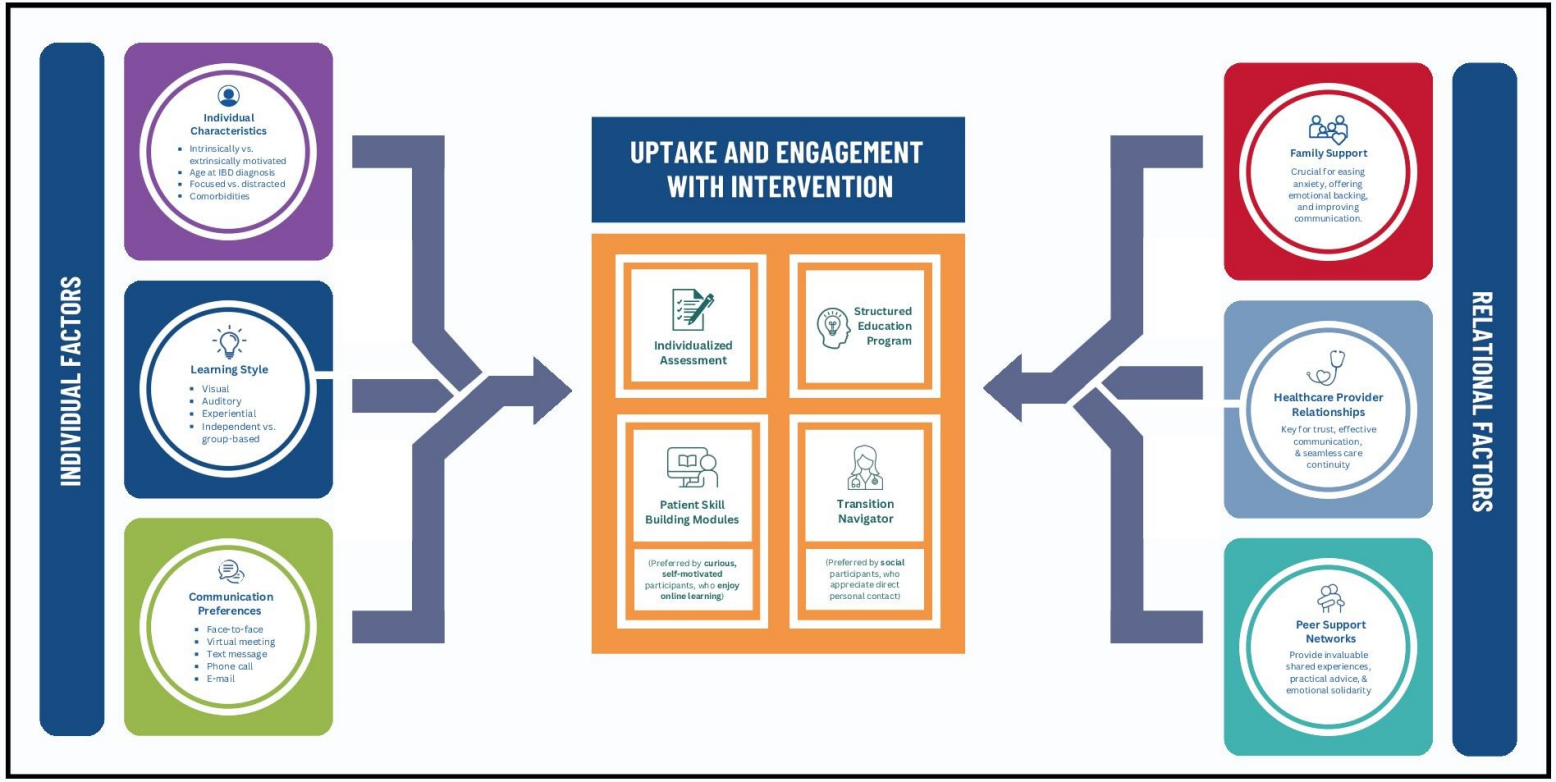
Factors influencing engagement with the intervention. IBD: inflammatory bowel disease.

Given the myriad of psychosocial stressors and challenges inherent in transitioning from pediatric to adult care, adolescents and young adults voiced that validation and emotional support were important. Participants in the intervention arm found these types of support in different intervention components based on their individual characteristics and relational factors (Figure 3).

Most adolescents and young adults who were social found that having direct access to a transition navigator (eg, via text or email) offered reassurance and interpersonal connection; one such adolescent or young adult shared: “100% talking to [my transition navigator is the most helpful component of the intervention] because I’m a people person, so talking to somebody is very helpful, whereas I just get bored doing the modules and stuff.” [I-016]. Other participants with similar personality characteristics felt that having a transition navigator helped foster a trusting relationship that extended beyond clinical interactions, providing them with an open outlet to voice their hopes, fears, and concerns regarding their transition to adult care. Many adolescents and young adults even felt immediately more prepared to transition, knowing that their transition navigators represented a dependable source of information that they could turn to and confide in during times of need: “I did get a person [transition navigator] who I can text about anything I’m struggling with. They’ve definitely helped me be open about how I’m feeling.” [I-007]

Other adolescents and young adults voiced that the variety of concepts covered in the skills-building modules validated their concerns and helped them feel less alone in coping with the challenges of living with IBD: “if they’re making modules like this, I’m not the only one that’s feeling like this.” [I-020]. Regardless of their personality traits, most adolescents and young adults described being well supported by their transition navigator in making informed decisions, whether that be related to accessing postsecondary accommodations or scholarships, obtaining referrals to relevant resources (eg, mental health services, volunteer opportunities, or support groups), or learning about the expectations of adult IBD providers.

#### Confidence and Independence

Adolescents and young adults outlined how the intervention promoted confidence in preparation for adult care. Some adolescents and young adults viewed the skills-building modules as the most relevant and beneficial aspect of the intervention because they gained independence through acquiring new strategies. Participants who were self-motivated and enjoyed online learning appreciated the wealth of information offered on a range of topics and the ability to access the modules at their own pace given their busy schedules at this stage of life (eg, planning for postsecondary education, participating in extracurricular activities, or working). Additionally, most adolescents and young adults felt that the skills and knowledge developed through the intervention could be applied to their illness and beyond: “I feel like the skills are the most applicable to your everyday life. You can use them with Crohn’s or without Crohn’s. They teach you how to deal with any obstacle you might have in that sense.” [I-019]

Many adolescents and young adults outlined the impact of the intervention in its entirety on their feelings of confidence and preparedness for a new care environment:

To have a navigator or to have modules, I just feel more comfortable now and more confident. Because even before I was like, “what’s going to happen when I turn 18? I don't want to leave [pediatric hospital] because I just feel like they know all about everything I've been going through and have helped me.”

[I-020]

While all participants receiving the intervention approved of its impact on their knowledge, skills, emotional well-being, confidence, and overall preparedness for transitioning to adult care, each adolescents’ and young adults’ preferences and personality traits impacted how and where they experienced the greatest benefit.

#### Barriers and Facilitators to Engagement

As outlined in Figure 3, a series of individual, intervention-specific, and familial characteristics supported or hindered adolescents’ and young adults’ engagement with the intervention and in their overall health care.

Individual characteristics that facilitated engagement included being newly diagnosed with IBD, having strong relationships with health care providers, and feeling well supported by family members and peers. Adolescents and young adults with recent diagnoses were motivated to complete the skills-building modules because they were interested in developing a higher-level understanding of their illness, including symptom management, self-care, and peer support. Adolescents and young adults who were intrinsically motivated and had learning styles conducive to online learning with visual aids, audio, and text-based materials favorably viewed the presentation of the modules. Consequently, these individuals did not require much prompting as they were driven to learn and expand their skill sets of their own accord.

The support systems adolescents and young adults had in place were critical to the uptake of the intervention, particularly for those who described themselves as forgetful. Most participants benefited from the navigator prompting or reminding them to complete the modules and appreciated the navigator’s persistence in communicating with them. Adolescents and young adults with comorbidities (eg, diabetes, arthritis, or anxiety) seemed to require more encouragement from the transition navigator or family members to support module completion, given they were managing multiple illnesses, appointments, and medication regimens. Some adolescents and young adults described completing the modules alongside parents who were interested in learning and supporting their adolescents or young adults, aiding participants who may otherwise have lacked interest in engaging with the content: “My mom was super on top of it [skills-building modules], and she’s like, ‘come do the modules with me’” [I-020]. When these supports were available, adolescents and young adults seemed better able to complete the materials and solidify their learnings through conversation and questions.

Regarding barriers to engaging with the intervention and completing the skills-building modules, most adolescents and young adults cited competing demands (eg, examinations, work, family, or romantic relationships) and forgetfulness as factors influencing their ability to commit time toward modules, despite their interest in the content. Some participants felt it challenging to initiate contact with the navigator or begin a new module without prompting because the research was admittedly not their top priority:

Sometimes, it’s hard for me to open the tablet and go on there [to do the modules] because it’s not the first thing that comes to my mind. Every day, I wake up, and I'm like, “Okay, I have to do this and that today. I also have to message this person [transition navigator],” and I don't get around to doing it.

[I-021]

The length of the modules was described by several participants as a deterrent, especially for those with difficulty staying on task. Some adolescents and young adults struggled to remain engaged and interested in the skills being discussed, inadvertently leading them to become distracted:

With the modules, I get distracted very easily, so it’s been very hard for me to stay on task. I’ve been struggling to remember since the end of grade 11 to do these modules. It’s not that they're boring per se, it’s that I'm not focused enough, ever.

[I-016]

Finally, adolescents and young adults whose learning preferences were at odds with how information was conveyed in the modules and educational curriculum faced barriers to retaining knowledge. For instance, participants who were visual learners expressed difficulties taking in the information and solidifying their learning, given that most of the content was delivered via a speaker on camera without visuals to illustrate concepts. This led to feelings of frustration and a lack of motivation to stay engaged for some:

I don’t know if it’s because I’m a visual person, but sometimes it’s really hard for me to sit down and watch somebody talk for 20 minutes. Something about it disengages me. My ears are open, but I'm not really taking in everything.

[I-021]

In summary, each adolescent’s or young adult’s learning styles, needs, and preferences were important factors to consider when using and evaluating the intervention.

### Theme #3: Considerations for Future Transition Care

#### Overview

Adolescents and young adults in both arms of the trial highlighted a “gentle transition,” defined as a period of overlap between pediatric and adult IBD providers or a joint visit with pediatric and adult providers, as an approach that would help reduce stress and illustrate that “the first team trusts the second team fully” [C-006]. Most participants also felt the topic of transition should be raised, and the process started early (around being aged 16 years) to allow ample time for gradual skill acquisition:

I’d say [introduce transition] at about 16 because 18 is when transition starts and throughout the journey, you don't really realize how little time you have until it’s right there and it’s knocking on your doorstep. But if you start teaching what they need to learn at around 16, I think they'll be able to develop skills and become more mature and be able to deal with those things as time goes on.

[I-019]

Adolescents and young adults approved of the value of a clinician (eg, transition navigator) responsible for “guiding [youth] through the transition process” [C-006], offering advice, addressing transition-related concerns, and providing service navigation. They felt having a trusted person they could go to (as opposed to a website or app) would allow them to practice the communication and advocacy skills needed for adult care in real time:

If you can have someone that people can reach out to and talk to, actually face-to-face whether it’s Zoom, texting, calling, or emailing, I feel like that would be beneficial just so they know they're actually talking to someone. And that would help with socializing and getting them used to adult responsibilities and communicating on their own.

[C-013]

Others suggested providing adolescents and young adults with case scenarios that commonly arise for transition-age youth with IBD to role play, followed by discussions about how to respond to different situations. They felt this approach would help them solidify experiential knowledge, build confidence, and prepare them for possible challenges:

I think it would be the most helpful to have [youth] actually do a fake phone call. Because if you're talking about something like a conversation, it’s better to learn it in its application and learn to try to say things yourself rather than knowing that you have to do something like, “oh, I *should* say that, I *should* do this”. Being forced to deal with some of the discomfort that might come along with the conversations and practicing that would be beneficial.

[C-036]

Finally, participants advocated for group-based education sessions (either in person or online) to support their learning. Those in the intervention arm felt that providing a method for adolescents and young adults with IBD to (optionally) interact with one another through the online platform would allow them to feel more connected and less alone.

#### Recommendations for Refinement of Biopsychosocial Intervention

Adolescents and young adults in the intervention arm highlighted the importance of keeping participants engaged in the intervention (and their care more broadly) around the time of transition when young people face competing priorities; thus, they suggested shortening the modules into “bite-sized” [I-007] pieces to improve their delivery. Several adolescents and young adults were interested in audio versions of the skills-building modules based on their learning styles and preferences. Many participants thought including games, knowledge tests, or offering awards for completion could help participants stay motivated and on task. Most participants were satisfied with the range of topics available regarding the module content. However, a few adolescents and young adults felt having materials focused on accepting IBD and adjusting to life with a chronic illness would be beneficial to them, especially for those newly diagnosed or experiencing challenges coping with IBD. Others suggested incorporating education focused on diversity within IBD, including the roles of faith, cultural traditions, and food customs in IBD management. Lastly, some adolescents and young adults felt the transition to postsecondary (education or employment) should be elaborated upon in the modules based on their experiences, especially advocating for themselves with teachers or employers and accessing accommodations.

## Discussion

### Principal Findings

Using qualitative methods, this study offered a detailed understanding of the experiences, priorities, and needs of adolescents and young adults with IBD during the pediatric-adult transition. Learning about the biopsychosocial intervention from adolescents’ and young adults’ perspectives contextualized the RCT results and provided insights about engagement, acceptability, and future directions. Our findings revealed the importance of making meaning during health care transition, the impact of the biopsychosocial transition intervention, and adolescents’ and young adults’ viewpoints on future transition care.

Regarding conceptualizations of the transition to adult care, adolescents and young adults with IBD expressed various ideas about its meaning. Hislop et al [25] outlined a similar range of views regarding the transition to adult care from youth with chronic conditions, from “laid back” to “anxious.” Additionally, the youth in their study expressed the value of social interaction with family, peers, and professionals to assist with the transition from pediatric to adult care [25]; findings echoed in the present study. Of note, most participants in our study had not yet transferred to adult care at the time of data collection, possibly influencing their conceptualizations of transition. Understanding adolescents’ and young adults’ views on the meaning of transition and the quality of their interpersonal relationships could support the development of personalized transition plans in clinical settings that consider their readiness. It is also important to examine whether adolescents’ and young adults’ conceptualizations of transition before they enter adult care impact their capacity for self-management and experiences posttransfer.

This study validates the importance of relational support for adolescents and young adults with IBD, which helped facilitate engagement with the intervention and readiness for transition. Fostering positive relationships with health care providers, family members, peers, or community members may play an important role in promoting flourishing, a known predictor of physical and mental health [26,27]. Given adolescents and young adults are often experiencing multiple life transitions simultaneously, external support is critical [26]. Future research could explore the development and evaluation of companion modules or educational resources for family members supporting adolescents and young adults with IBD.

Tailoring educational resources and support to align with the needs of adolescents and young adults arose as a prominent concept in this study. This included considering adolescents’ and young adults’ communication preferences, learning styles, familial or community supports, and pre-existing traits, including age at IBD onset and personality characteristics, in designing and delivering patient education. Individualizing care according to genetic, social, and individual- and family-specific factors is a key tenet of precision child health, an emerging paradigm for pediatric quality and safety [28]. Precision child health focuses on the unique needs and characteristics of pediatric patients and their families to provide proactive, person-centered care [28]. Moreover, offering personalized support for adolescents and young adults with chronic conditions transitioning to adult care according to their level of preparedness has been endorsed in the literature. Charles et al [29] identified a typology of transition readiness for adolescents and young adults with congenital heart disease, suggesting that different groups require varying levels of support in preparation for the transition, from minimal intervention required to “follow-up needed” to “at-risk.” A transition intervention that is customized based on the ethnocultural needs of adolescents and young adults with traumatic brain injury has also been described and reported [30]. Further, the IBD literature demonstrates the importance of delivering culturally sensitive care that recognizes the impact of health inequities, cultural values and beliefs, and the roles of implicit or explicit biases on patients’ experiences navigating the health care system [31]. Our results provide evidence of the criticality of attending to adolescents-, young adults-, family-, and system-level factors in providing transition care to promote optimal experience, uptake, engagement, and outcomes.

Patient navigators have been identified as a promising intervention for adolescents and young adults with chronic conditions transitioning to adult care [32,33]. Our results confirmed the value and acceptability of the relational support offered by a transition navigator, with participants detailing the navigator’s impact on their experiences and outcomes during the transition. This was especially apparent for adolescents and young adults with challenging diagnostic journeys, mental health concerns, and co-occurring conditions. This speaks to the potential usefulness of a transition navigator intervention for adolescents and young adults with chronic health conditions or mental health conditions more broadly. Given the logistical challenges of holding joint pediatric-adult clinic appointments in North America, cross-appointed navigators could be a favorable option for promoting continuity of care [34,35]. Further exploration of individual characteristics, including personality traits and existing supports, and how these relate to engagement during transition is warranted. Adolescents and young adults in our study expressed their interest in gamified transition resources to enhance motivation to engage with educational materials. Indeed, a recent scoping review identified that gamification improves patient engagement and biopsychosocial outcomes and could represent a valid approach to cancer patient education [36]. Future research could explore gamification within IBD care and its impact on patient experience, knowledge, and outcomes. Finally, given the complexity of factors (eg, communication preferences, personality, or learning styles) influencing adolescents’ and young adults’ transition needs, future iterations of the biopsychosocial transition intervention could consider the integration of artificial intelligence to support adolescents and young adults. This would enable tailored guidance when navigator support is unavailable (eg evenings or weekends) or when adolescents and young adults prefer more self-directed approaches.

### Limitations and Future Directions

This study’s results should be considered in light of some limitations. Sampling bias could be a concern given that the qualitative study participants were enrolled in an RCT and consisted of mostly female participants. Regarding transferability, our findings may not apply to all adolescents and young adults with IBD or those in different health care systems or regions. Additionally, only 1 participant had completed any education modules at the time of the interview, so we could not assess participants’ perceptions of this aspect of the intervention. To enhance trustworthiness, we used member checking with the YAP. YAP members reviewed and confirmed the data and interpretations, which helped ensure our findings’ accuracy. Following initial coding and analysis based on their lived experiences, they provided their insights and perspectives. Finally, they co-authored, reviewed, and offered feedback on this paper.

Future research in this field could examine the experiences of adolescents and young adults with IBD in various cultural and socioeconomic contexts. This would allow for a more complete understanding of the transition process and different groups’ unique challenges given research has shown that access to resources, cultural values, health literacy, family roles, faith, and stigma play important roles in the daily realities of individuals with IBD [31,37]. Qualitative research involving data collection with adolescents and young adults at multiple time points (eg, pretransfer, during the transition, or posttransfer) could offer unique insights into how their care needs and priorities evolve to support the development of tailored interventions. Finally, the development and evaluation of resources targeting caregivers and parents of adolescents and young adults with IBD could be considered, given their critical roles in mentoring and coaching adolescents and young adults during transition.

### Conclusions

Incorporating qualitative data within the RCT enabled a multifaceted exploration of the acceptability of a biopsychosocial transition intervention through different sources of information to support the translation of findings into practice. Our findings provide important insights into the needs and experiences of adolescents and young adults with IBD during the transition, including the criticality of considering individual, family, and system-level factors when implementing clinical interventions for this population. These results will contribute to developing youth-friendly resources and possibly refining the biopsychosocial transition intervention before scale and spread.

## Abbreviations:

C: control arm participant
I: intervention arm participant
IBD: inflammatory bowel disease
RCT: randomized controlled trial
YAP: Youth Advisory Panel
